# Complete monoplegia due to limb-kinetic apraxia in a patient with traumatic brain injury: A case report

**DOI:** 10.1097/MD.0000000000022452

**Published:** 2020-12-04

**Authors:** Sung Ho Jang, You Sung Seo

**Affiliations:** Department of Physical Medicine and Rehabilitation, College of Medicine, Yeungnam University 317-1, Daemyungdong, Namku, Taegu, Republic of Korea.

**Keywords:** corticofugal tract, diffusion tensor tractography, limb-kinetic apraxia, monoplegia, traumatic brain injury

## Abstract

**Rationale:**

Limb-kinetic apraxia (LKA) is a disorder of movement execution that is a result of injury to the corticofugal tracts (CFTs) from the secondary motor area. We report on a patient with traumatic brain injury (TBI) and complete monoplegia due to LKA, which was mainly ascribed to injury of the CFT from the secondary motor area using diffusion tensor tractography.

**Patient concerns:**

A 35-year-old male was struck by a car from the side during riding an autocycle and received direct head trauma as a result of falling to ground. He lost consciousness for approximately 1 month and experienced continuous post-traumatic amnesia after the accident. The patient's Glasgow Coma Scale score was 3 and he showed quadriparesis including complete monoplegia of his left arm since the onset of TBI.

**Diagnoses:**

The patient diagnosed complete monoplegia due to LKA after traumatic brain injury.

**Interventions:**

He underwent conservative management for TBI followed by rehabilitation at approximately 2 months after onset. Outcomes: At 32-month after onset, weakness on left arm (Manual Muscle Test [MMT]:0) and partial weakness of left leg (MMT:3).

**Outcomes:**

Results of electromyography and nerve conduction studies of left extremities were normal. Motor evoked potential values obtained from the abductor pollicis brevis muscle (APB) were: right APB latency 22.3msec, amplitude 1.6mV; left APB latency 22.8msec, amplitude 1.5mV. After 2 weeks of administration of dopaminergic drugs for improvement of LKA, left arm weakness had recovered to level that permitted movement against gravity (MMT:3). Diffusion tensor tractography at 32-month after onset showed right corticospinal tract discontinuation at the pontine level and partial tearing of the left corticospinal tract at the subcortical white matter. In addition, the left CFT from the supplementary motor area showed partial tearing at the subcortical white matter.

**Lessons:**

The LKA due to injury of the left supplementary motor area-CFT was demonstrated in a patient with complete monoplegia following TBI. Accurate diagnosis of LKA is important for successful rehabilitation because LKA is known to respond to dopaminergic drug treatment.

## 1 Introduction

Limb-kinetic apraxia (LKA) is a disorder of movement execution that is a result of injury to the corticofugal tracts (CFTs) from the secondary motor area.^[1–3]^ LKA can be diagnosed by observing the characteristic movement pattern of LKA: awkward, clumsy and coarse with mutilated execution of simple movements.^[1–3]^ The introduction of diffusion tensor tractography (DTT), which is reconstructed from diffusion tensor imaging data, has allowed 3-dimensional estimation of the connectivity of the CFTs from the secondary motor area.^[4]^ By using DTT, several studies have demonstrated LKA due to injury of the CFTs from the secondary motor area in patients with brain injury.^[5–15]^ The patients in those studies showed partial weakness and characteristic LKA movement patterns. However, no study of a patient with complete monoplegia of a specific limb due to LKA following injury of the CFTs has been reported.

In this case study, we report on a patient with traumatic brain injury (TBI) who exhibited complete monoplegia due to LKA, which was mainly attributed to injury of the CFT from the secondary motor area, as demonstrated by using DTT.

## 2 Case report

**Figure 1:**
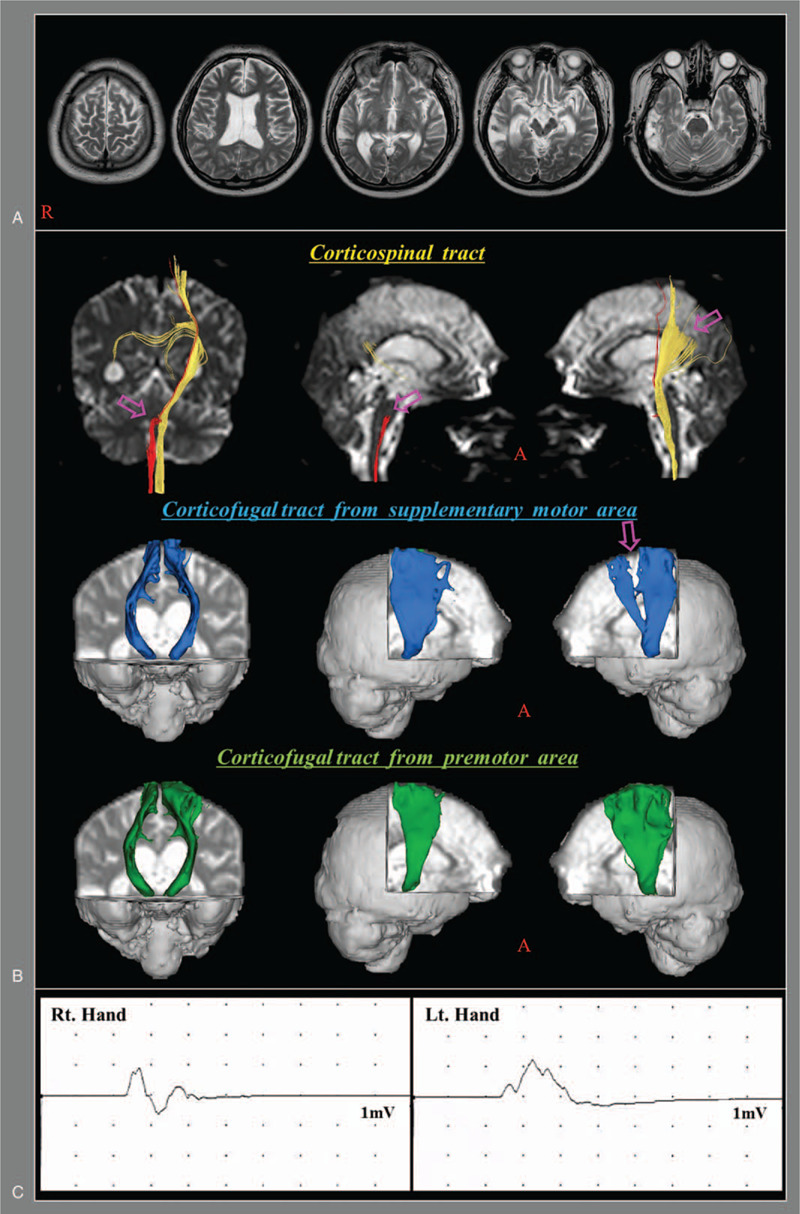
A. T2-weighted brain magnetic resonance images obtained at 32 months after onset show a leukomalactic lesion in the right temporal lobe. B. On 32-month diffusion tensor tractography, the right corticospinal tract has a discontinuation at the pontine level (arrow), whereas the left corticospinal tract shows partial tearing at the subcortical white matter (arrow). The corticofugal tract from the left supplementary motor area shows partial tearing at the subcortical white matter (arrow). C. Motor evoked potentials obtained from abductor pollicis brevis (APB) and tibialis anterior (TA) muscles (right side APB latency: 22.3 msec, amplitude: 1.6 mV; left side APB latency: 22.8 msec, amplitude: 1.5 mV).

A 35-year-old male with no history of neurological, physical, or psychiatric illness suffered head trauma resulting from a car accident. He was struck by a car from the side while riding an autocycle and received a direct head trauma by falling to the ground. He lost consciousness for approximately 1 month and experienced continuous post-traumatic amnesia after the accident. The patient's Glasgow Coma Scale score was 3 when he arrived at a hospital. Initially, he underwent conservative management for traumatic intracerebral hemorrhage in the right temporal lobe, subdural hemorrhages in both frontal lobes, and diffuse axonal injury. He showed quadriparesis including complete monoplegia of his left arm since the onset of TBI. At approximately 2 months after onset, he underwent rehabilitation. When admitted to the rehabilitation department of our university hospital at 32 months after onset, he had complete weakness of his left arm (Manual Muscle Test [MMT]: 0), partial weakness of his left leg (MMT: 3^−^), and mild weakness of his right arm and leg (MMT: 4). He did not exhibit any movement difficulties when he performed movements of his right arm and leg. Brain magnetic resonance imaging (MRI) revealed a leukomalactic lesion in the right temporal lobe (Fig. 1-A). Results of electromyography and nerve conduction studies of his left upper and lower extremities were normal. Motor evoked potential results obtained from the abductor pollicis brevis muscle (APB) and tibialis anterior muscle (TA) were as follows: right side APB - latency 22.3 msec, amplitude 1.6 mV; left side APB- latency 22.8 msec, amplitude 1.5 mV; right side TA - latency 31.2 msec, amplitude 2.4 mV; and left side TA - latency 31.7 msec, amplitude 0.2 mV (Fig. 1-C).^[16]^ After 2 weeks of administration of dopaminergic drugs for improvement of LKA (ropinirole, 3 mg; bromocriptine, 10 mg; and levodopa, 375 mg), the weakness of his left arm had recovered to a level that allowed movement against gravity (left shoulder flexor MMT: 3; and left elbow flexor MMT: 3).^[5,6,17]^ The patient has provided informed consent for publication of the case and the study protocol was approved by the institutional review board of a university hospital.

### 2.1 Diffusion tensor imaging

Diffusion tensor imaging data were acquired 2 years after the TBI by using a 6-channel head coil on a 3 T Philips Gyroscan Intera (Philips, Best, Netherlands) with single-shot echo-planar imaging. For each of the 32 non-collinear diffusion-sensitizing gradients, 70 contiguous slices were acquired parallel to the anterior commissure–posterior commissure line. Imaging parameters were as follows: acquisition matrix = 96 × 96; reconstructed to matrix = 192 × 192 matrix; field of view = 240 mm × 240 mm; TR = 10,398 ms; TE = 72 ms; parallel imaging reduction factor (SENSE factor) = 2; EPI factor = 59; b = 1000 s/mm^2^; NEX = 1; and slice thickness = 2.5 mm (acquired isotropic voxel size 2.5 mm × 2.5 mm × 2.5 mm).

Fiber tracking was performed by using functions within the Oxford Centre for functional magnetic resonance imaging of the brain software library (www.fmrib.ox.ac.uk/fsl). Affine multi-scale 2-dimensional registration was used to correct for head motion effects and image distortion due to eddy currents. Fiber tracking was performed by applying a probabilistic tractography method based on a multifiber model and was applied by utilizing tractography routines implemented in functional magnetic resonance imaging of the brain Diffusion software (5000 streamline samples, 0.5 mm step lengths, curvature thresholds = 0.2).^[18]^ For corticospinal tract (CST) analysis, the seed region of interest (ROI) was placed on the portion of the CST at the anterior pontomedullary junction on the color map, and the target ROI was placed on the primary motor cortex (anterior boundary — precentral sulcus, posterior boundary — central sulcus, medial boundary — midline between the right and left hemispheres, lateral boundary — a line horizontal to the midline passing through the lateral margin of the precentral knob).^[19]^ For analysis of the CFTs from the dorsal premotor cortex (dPMC; dPMC-CFT) and the supplementary motor area (SMA; SMA-CFT), the seed ROIs were placed on the crus cerebri on the fractional anisotropy map. The target ROIs were placed on the dPMC (anterior boundary — the line joining the anterior extent of the SMA, posterior boundary — precentral sulcus, medial boundary — the lateral margin of the SMA, lateral boundary — the line horizontal to the midline passing through the lateral margin of the precentral knob) and the SMA (anterior boundary — the line drawn through the anterior commissure perpendicular to the anterior commissure–posterior commissure line, posterior boundary — anterior margin of the primary motor cortex, medial boundary — midline between the right and left hemispheres, lateral boundary — the line 15 mm lateral from the midline).^[19–21]^

On DTT at 32 months after onset, reconstruction of the right CST revealed a discontinuation at the pontine level, whereas the left CST revealed partial tearing at the subcortical white matter (Fig. 1-B). The left SMA-CFT showed partial tearing at the subcortical white matter.

## 3 Discussion

In this study, we describe a patient with complete weakness of his left arm following a TBI. Based on our observations, we ascribe the complete weakness of his left arm mainly to LKA for the following reasons. First, because the patient did not have difficulty in understanding and performing movements, we were able to rule out ideational and ideomotor apraxia. Second, although the right and left CSTs showed discontinuation and partial tearing, respectively, the motor evoked potential latencies of muscles in both hands were normal (although the amplitudes were mildly low); thus, we concluded the integrities of both CSTs were preserved, suggesting that the DTT results for both CSTs were indicative of a type of diffuse axonal injury (DAI).^[16,22]^ In addition, we ruled out peripheral nerve injury because the electromyography and nerve conduction results for the left arm revealed no abnormality. However, we did observe injury (partial tearing) of the left SMA-CFT on DTT. DTT is known to have unique advantage to find axonal lesion in TBI without definite abnormality on conventional brain MRI.^[12–15]^ Considering his brain MRI finding (no abnormality through the pathway of the left SMA-CFT), DTT finding of the left SMA-CFT, and the duration of loss of consciousness (approximately one month), DAI appeared to be the main pathophysiological mechanism of his left SMA-CFT injury.^[11–15]^ Third, the patient showed rapid motor recovery of his left arm weakness with the administration of dopaminergic drugs, which are known to be effective in the treatment of LKA.^[5,6,17]^ Therefore, we concluded that the complete weakness of his left arm could be ascribed to LKA mainly resulting from DAI of the SMA-CFT and partly from DAI of the right CST.

In conclusion, LKA due to injury of the left SMA-CFT was demonstrated in a patient with complete monoplegia following TBI. To our best knowledge, this study is the first study to report a patient with monoplegia due to LKA. Accurate diagnosis of LKA is important for successful rehabilitation because LKA is known to respond to dopaminergic drug treatment.^[5,6,17]^ Regarding monoplegia in patients with TBI, clinicians usually suspect severe injuries of the peripheral nerves. Therefore, our results suggest the need to evaluate the CFTs from the secondary motor area in patients with unexplained monoplegia following TBI.

## Author contributions

**Conceptualization:** Sung Ho Jang.

**Data curation:** You Sung Seo.

**Methodology:** You Sung Seo.

**Writing – original draft:** Sung Ho Jang.

**Writing – review & editing:** Sung Ho Jang.
